# A randomized controlled trial comparing nerve block and mandibular infiltration techniques in posterior mandible implant surgeries

**DOI:** 10.4317/jced.54330

**Published:** 2018-10-01

**Authors:** Matias Garcia-Blanco, Ariel-Felix Gualtieri, Sebastian-Ariel Puia

**Affiliations:** 1 Universidad de Buenos Aires. Facultad de Odontología. Cátedra de Cirugía y Traumatología Bucomaxilofacial I. Buenos Aires, Argentina; 2 Universidad de Buenos Aires. Facultad de Odontología. Cátedra de Biofísica y Bioestadística. Buenos Aires, Argentina

## Abstract

**Background:**

To compare global surgical pain under nerve block and mandibular infiltration anesthesia techniques, and to evaluate pain during drilling and the distance to the mandibular canal in posterior mandible implant surgeries.

**Material and Methods:**

A prospective, randomized, controlled, double-blind, clinical trial was conducted to compare nerve block (Group A) to mandibular infiltration (Group B) techniques for dental implant placement. Global surgical pain (VAS = visual analogue scale), pain during drilling or implant placement (MPQ = McGill pain questionnaire) and distance to the mandibular canal (Image J) were statically analyzed. Age, gender, anxiety levels, tooth to be replaced, implant size, adjacent teeth and duration of surgery were also analyzed.

**Results:**

172 patients were included and 283 dental implants were analyzed. VAS values were significantly higher in Group B (*p*<0.05). In Group A, 99% of the surgeries were performed painlessly during drilling and implant placement, but in Group B, 11.6% of implant placements (17 implants) felt pain during these surgical steps. Mean distance to mandibular canal (3.8 mm, range: 0.0 to 7.0) in those 17 implants placed under mandibular infiltration was clinically and statistically similar to the mean distance (3.0 mm, range: 0.0 to 9.0) of 130 implants placed painless (*p*=0.10). Pain during drilling under mandibular infiltration was significantly associated with the duration of surgery (*p*<0.05) and to both adjacent teeth being present (*p*<0.05).

**Conclusions:**

Although both techniques are safe and effective for placing implants in the posterior mandible, nerve block provides a more profound analgesia than mandibular infiltration. When placing implants under mandibular infiltration, as getting closer to the canal does not increase the feeling of pain, it is not recommended to use the presence of pain as a preventive resource to avoid inferior alveolar nerve injuries.

** Key words:**Dental implant, mandibular infiltration anesthesia, nerve block, pain, nerve injury.

## Introduction

Peripheral sensory nerves are essential in enabling people to interact with the environment because they provide sensations of touch, pressure, temperature change and pain (1). Speaking, eating, kissing, tasting and swallowing all depend on peripheral sensory nerves. It is thus important to consider that they could be injured during acute dental intervention (2,3).

Inferior alveolar nerve (IAN) and lingual nerve (LN) injuries could occur in surgeries such as wisdom teeth extractions, orthognathic procedures and dental implant placements. They could also occur during the application of routine nerve block anesthesia or during endodontic treatment (4-7). Patient symptoms include complete absence of sensation, diminished or increased sensitivity, abnormal sensations which may not be unpleasant, and spontaneous or mechanically evoked painful symptoms. Additionally, if the LN is involved, there may be absence or distorted gustatory perception (6-8).

The mandibular infiltration anesthesia technique has been proposed as an alternative to the IAN block for placing dental implants in the posterior mandible (9,10). Some articles suggest that because the IAN would not be completely anesthetized under mandibular infiltration, the painful stimulus would enable the patient to warn the surgeon before any nerve injury could be caused (10-12). This argument was not based on a randomized controlled trial (RCT), but if it is correct, painful cases would be higher during drilling near the mandibular canal (MC).

In contrast, another study observed that in edentulous mandibles the IAN is found as a single trunk in only 3.6% of the cases, being much more likely to present a different kind of plexus (13). It may also be possible to mechanically stimulate a nerve branch located far from the central trunk. One clinical trial was presented on two patients in whom pain occurred 2.9 and 6.7 mm away from the MC (14). Another study reported completely painless surgery under mandibular infiltration technique for an implant in contact with the MC, in which paresthesia developed (15).

The aims of this RCT were:

-To compare global surgical pain in dental implant surgeries performed under nerve block and mandibular infiltration.

-To evaluate the association between pain during drilling or implant placement and distance to the MC in dental implant surgeries performed under nerve block and mandibular infiltration.

## Material and Methods

* Study Population 

This was a prospective, randomized, double-blind (patient and operator), parallel group comparison clinical trial, conducted at the Department of Oral Surgery I of the School of Dentistry of the University of Buenos Aires (Argentina), from January 2013 to December 2016, on patients requiring implant-prosthetic rehabilitation in the posterior mandible.

Anesthesia techniques for surgeries were previously randomized using random numbers function in software (Microsoft Office Excel 2007, Redmond, CA) and kept inside envelopes numbered according to the order in which patients presented for surgery. The patients and evaluators were blinded to the group assignments. Group A patients received implants under nerve block technique, and Group B patients received implants under mandibular infiltration technique.

This research was performed following the recommendations of the Consort Statement for reporting RCTs and the ethical principles of the Helsinki Declaration regarding research on humans. Accordingly, all patients provided written informed consent prior to surgery in order to take part in the trial. The study design was approved by the Ethical Committee of the University of Buenos Aires (Research Ethics Committee No. 53-2013). This study did not receive industrial funding.

* Selection Criteria 

Inclusion criteria for this study were.

- Age > 18 years.

- Dental implant placement for inferior first and second molars, and inferior first and second premolars.

- Single or multiple dental implant surgeries.

- Healed alveolar ridges.

- Signature of informed consent form.

Exclusion criteria were:

 - Immediate dental implant placement.

- Dental implant placements including any bone grafting procedures.

- Medical conditions contraindicating implant surgery.

- Patient unable to receive any standard medications.

- Administration within 48 hs. of any analgesic or sedation drug.

- Patients able to identify the anesthesia technique administrated (e.g. dentists).

Implant surgeries were excluded from analysis if:

- There were two consecutive failed anesthesia applications, 

- Surgery lasted more than 90 minutes.

* Surgical Procedure 

All patients were specifically informed that the main objective was to provide painless surgery, and that if they felt pain, immediate anesthesia reinforcement would be provided. Clinical examination, evaluation of local and systemic factors, panoramic radiograph and CT scan were used to plan surgery. Patients received 500mg amoxicillin (Amixen, Laboratorio Bernabo, Argentina) or 500mg azithromycin (Misultina, Laboratorio Bernabo) 1 hour before surgery (16). Anesthesia technique was administrated by a single operator (first author) in absence of the surgeon, to blind both patient and operator. Operators were previously calibrated department residents and professors. The envelope containing the anesthesia technique was opened before each surgery. The anesthetic drug was 4% articaine with 1:100,000 adrenalin (Totalcaina Forte, Laboratorio Bernabo, Argentina).

IAN block (Group A) was performed using the conventional Halstead approach with a 27-G long needle by touching the bone and retrieving the needle for the IAN and another withdrawal for the LN providing 1.8ml of anesthetic solution (17). After block confirmation, a supplemental buccal infiltration anesthesia of 0.9ml for each implant to be placed was provided for the buccal nerve (BN).

Mandibular infiltration technique (Group B) was done using a 27-G long needle to place 1.8ml in the buccal vestibule and another 0.9ml in the lingual vestibule, and finally 0.3ml under the lingual periosteum to help to raise the flap (10). For each additional implant in the area, 0.9ml and 0.3ml were placed in the buccal and lingual vestibules, respectively.

If anesthesia was not achieved after two approaches, the patient received intervention but was excluded from analysis. After anesthesia was confirmed, the operator entered the room and began the surgery. All surgical interventions consisted of raising a muco-periosteal flap, drilling with copious cooling at 800 - 1200 rpm, manual and instrumental implant placement (Q-Implant, Buenos Aires, Argentina), and primary closure of the wound (18). When surgeries concluded, intraoral radiographs were taken using paralleling technique, and the blinded operator completed the research data sheet. All patients received post-operative antibiotics (amoxicillin or azithromycin) and analgesic drugs (ketorolac or ibuprofen).

* Data Compilation

All information was recorded on the day of the surgery. No drop-out was recorded. A previously established standard protocol was used to compile the following data for all patients: age, sex, teeth to be replaced, implant length and diameter, adjacent mesial and distal teeth (present or absence), duration of surgery.

The following data and clinical parameters were registered.

**Figure 1:**
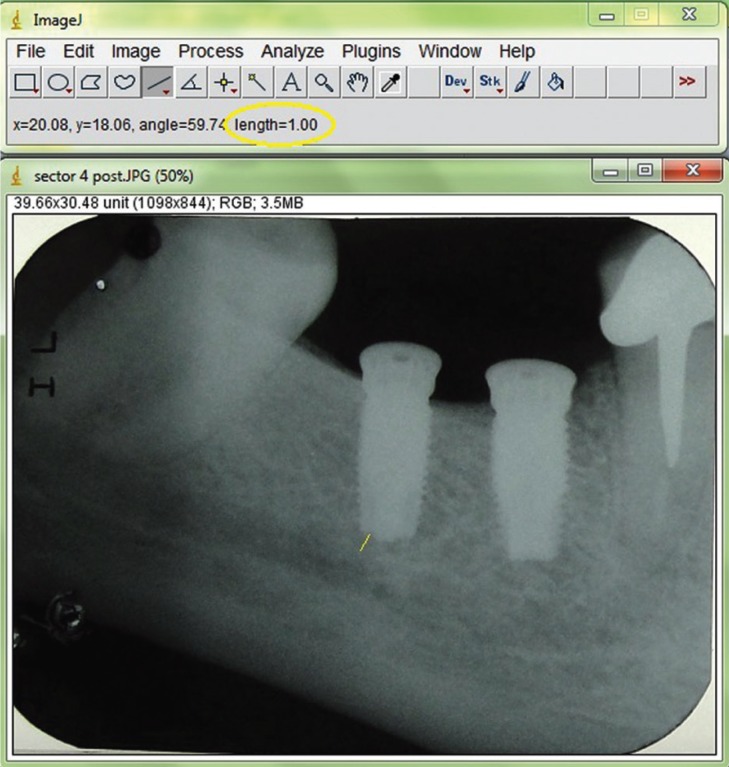
Measurement of distance to mandibular canal (Image J).

• Previous anxiety scale: before surgery, the Modified Dental Anxiety Scale (MDAS) was provided. It consists of 5 questions about anxiety level regarding normal dental treatment situations (score 5 to 25) (19).

• Global surgical pain (including anesthesia, incision, raising the flap, drilling, implant placement and wound closure): All patients were asked to report global surgical pain experienced throughout surgery on the visual analogue scale (VAS), which consists of a 10 cm line to evaluate the amount of pain, ranging from 0 (no pain whatsoever) to 10 (worst pain imaginable) (20).

• Pain during drilling or implant placement: if patient referred pain during these surgical steps, the Mc Gill Pain Questionnaire – Spanish Version (MPQ-SV) was completed (21). If the patient had no pain during these steps, MPQ-SV was considered zero. The MPQ-SV consists of various pain descriptions according to their pain quality and ranks the descriptions of a certain quality according to intensity. It consists of 66 pain descriptors organized in 19 subclasses, and within each subclass (sensory, affective and evaluative), descriptors are ranked in order of intensity. The pain rating index (PRI) value (range 0-66) is the sum of the intensity ranking of the chosen pain words.

• Distance from the bottom of the implant to the internal roof of the MC was evaluated on intraoral radiographs performed using paralleling technique immediately after the surgery concluded. Radiographs were scanned, digitized in JPG format with 300-dpi resolution, and stored in a personal computer. The distance was measured using Image J 1.42 software (National Institute of Mental Health, Rockville, Md) (22). Measurements were taken by a single blinded investigator who was not involved in the surgical procedure. Each image was calibrated using the known length of the implant (Fig. 1).

* Statistical Analysis 

Descriptive statistics of numerical variables included arithmetic mean, median, standard deviation (SD), minimum and maximum (range). Between-group comparisons were done by Wilcoxon rank-sum test. Correlation among MDAS and VAS was explored using the Spearman correlation coefficient. Non-parametric tests were performed because the data did not follow normal distribution as suggested by the Shapiro-Wilk test. Association between categorical variables was examined using Pearson Chi-square or Fisher exact test when there were expected frequencies under 5. The statistical analysis was performed with the software package Infostat version 2015. The level of significance was set at 0.05.

## Results

* Patient population

**Figure 2:**
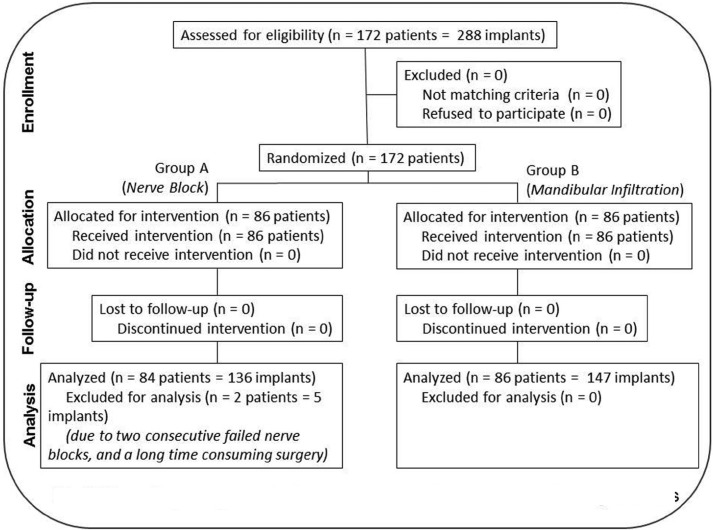
Flow diagram generated in accordance with CONSORT 2010 guidelines.

One hundred and seventy two consecutive patients were included in this study (73 males and 99 females). Mean age was 50 (SD 11) years. No patient withdrawal was recorded. Two patients in group A (IAN block group) received intervention but were excluded from analysis – one because complete anesthesia of IAN and LN was not achieved after two nerve blocks, and another because surgery lasted 105 minutes. One hundred and thirty-six (136) implants were placed in Group A (Nerve Block) and 147 in Group B (Mandibular Infiltration) (Fig. 2).

Sixty-six percent of the implants were 10mm long, and only 1.4% was longer than 12mm ( Table 1). No implant was placed intruding into the MC, but one nerve injury was recorded. The patient (female, 37 years old, MDAS=12) did not refer pain during surgery (MPQ-SV=0, VAS=0, distance to MC=1mm, duration of surgery =75 min), which was performed under nerve block. When patient phoned the surgical team in post-op, she reported nerve injury and was immediately scheduled for another visit. A post-surgical CT scan confirmed implant placement 1 mm above the MC, and showed low density bone surrounding MC. Lack of density around the MC is considered to be the cause of indirect nerve compression. The patient refused to have the implant removed because she wished to avoid another surgical intervention. Oral dexamethasone, ibuprofen and B vitamin complex were administered. The injury was monitored weekly, and full recovery was observed after 2 months. Prosthetic rehabilitation was uneventful.

**Table 1:**
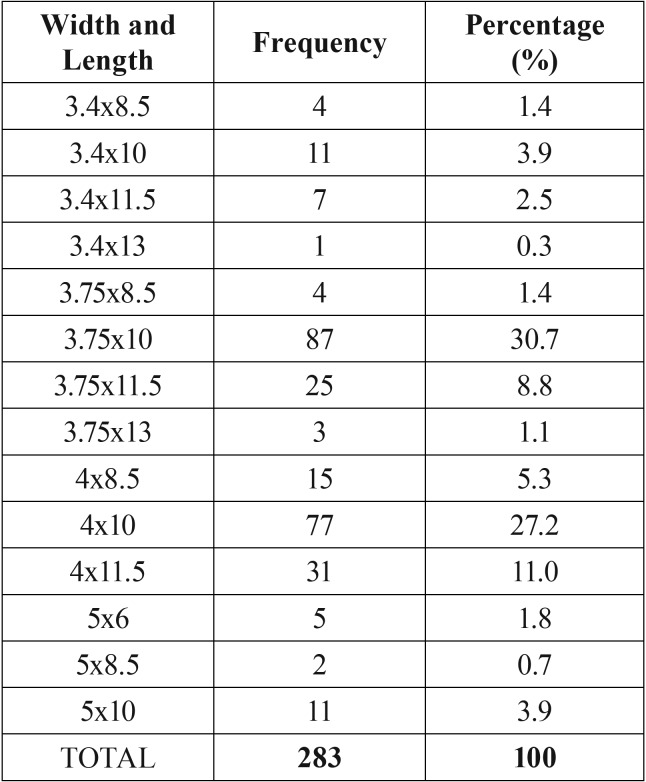
Implant width and length distribution for the whole sample.

* Clinical Data

GLOBAL SURGICAL PAIN (VAS)

Global pain (VAS) values were significantly lower in the group receiving nerve block (Group A) than in the group receiving mandibular infiltration (Group B) (*p*<0,05). The means were 0.4 (SD 0.8) (range = 0.0 to 4.0) and 0.7 (SD 1.1) (range = 0.0 to 5.0), respectively ( Table 2).

**Table 2:**
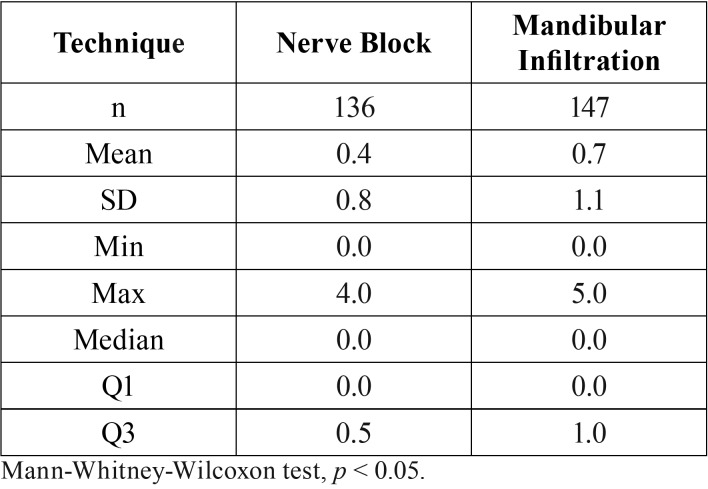
Description of global pain VAS values for nerve block and mandibular infiltration techniques.

The correlation between VAS values and anxiety levels (MDAS) was not statistically significant in the entire population (*p*=0.39), or in group A (*p*=0.10) or group B (*p*=0.72).

Comparison of VAS values between operators skills (residents and professors) showed no statistically significant difference for group A (*p*=0.09) or group B (*p*=0.22).

No correlation was found between VAS values and age (*p*=0.67) or gender (*p*=0.24), or tooth to be replaced (*p*=0.63), in the whole population.

PAIN DURING DRILLING OR IMPLANT PLACEMENT (MPQ-SV)

Group A (Nerve Block)

In group A, only one implant placement referred pain during drilling or implant placement. In this patient, the implant was placed at 4mm from the MC, MDAS was 21, duration of surgery was 65 min, MPQ-SV score was 10, and VAS was 4. All other 135 implants were placed in absence of pain during drilling and implant placement. Mean distance to MC was 3.00 (range: 0.0 to 8.5) ( Table 3).

**Table 3:**
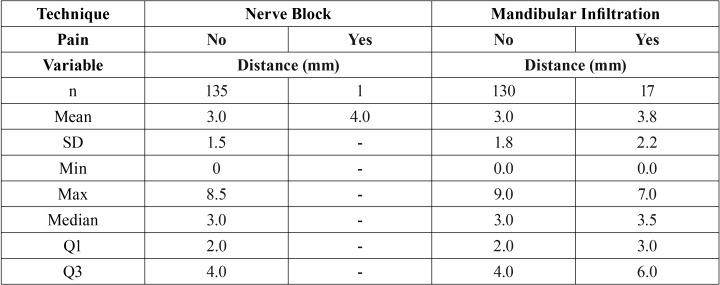
Description of distance to mandibular canal in painful and painless surgeries under nerve block and mandibular infiltration techniques.

Group B (Mandibular Infiltration)

In group B, 17 implant patients referred pain during drilling or implant placement, while 130 implants were placed in absence of pain during these steps. In the cases that referred pain, mean MPQ-SV was 11 (SD 4) and mean distance to MC was 3.8 (range: 0.0 to 7.0). Mean distance to MC in the group without pain was 3.0 (range: 0.0 to 9.0). No significant difference was observed between the two groups (*p*=0.10) ( Table 3). Comparison of VAS values between groups showed statistically significant differences, with higher VAS values in the group that felt pain (2.5 SD 1.2) than in the group without pain (0.5 SD 0.8) (*p*<0.05).

No significant association was found (*p* = 0.39) between pain during drilling or implant placement and the tooth to be replaced under mandibular infiltration.

Statistical association was found between the number of adjacent teeth and pain during drilling or implant placement under mandibular infiltration (*p*<0.05). Pain was more frequent when both adjacent teeth were present (30%), than when only one tooth or no tooth was present (7% and 11%, respectively).

**Figure 3:**
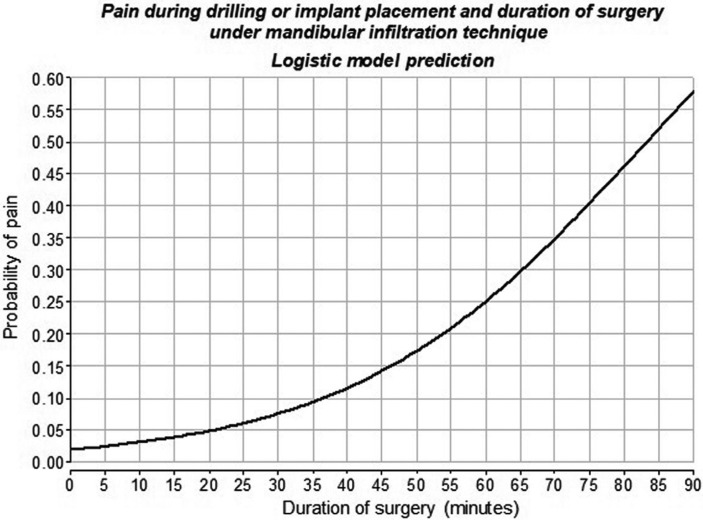
Relationship between the probability of pain during drilling or implant placement and duration of surgery under mandibular infiltration technique, according to a logistic model.

There was also significant association between duration of surgery and pain during drilling or implant placement under mandibular infiltration. The chance of presence of pain was significantly greater the longer the surgery lasted (*p*<0.05). This finding enabled a logistic model to be developed (Fig 3).

Pain during drilling or implant placement was variably described as acute, short-term, ceasing when stimulus ceased, low intensity, weak and bearable. Descriptions of temperature and electrical characteristics varied. In general, sensory, emotional, and evaluative appraisals of pain during drilling and implant placement were low (mean MPQ-SV was 11 (SD 4)). Out of 17 pain situations, 10 occurred only during drilling, 5 occurred only during implant placement, and 2 occurred during both steps. MPQ-SV mean (ranges) were 10 (6 to 16), 11 (6 to 16) and 18.5 (18 to 19), respectively.

The overall tendency of fewer cases of painful than painless surgeries during drilling or implant placement continued after a cutoff of the sample at the traditionally proposed safety zone (2mm) during the mandibular infiltration technique. Only 3 out of 33 surgeries in which implants were placed closer than 2mm to the MC referred pain during these steps. No significant association was found between distance to MC (<2mm or ≥2mm) and presence of pain (*p*=0.76).

## Discussion

IAN block and mandibular infiltration techniques are safe and effective for placing implants in the posterior mandible, median global pain VAS values of both techniques were zero, and all surgeries achieved their planned goals. The main advantage of nerve block is providing a statistically more profound analgesia, as reflected by VAS values in this study; and the main advantages of infiltration are its simple application, quick onset, and the fact that it cannot injure the IAN with the needle.

In this trial, patients reported intraoperative pain throughout surgery through the VAS. Mean VAS values were 0.4 (SD 0.8) in the nerve block group and 0.7 (SD 1.1) in the mandibular infiltration group. These results are similar to the mean VAS values reported in other studies of posterior mandible implant surgeries: 1.03 (SD 0.83) (23), 1 (SD 2.73) (24) and 0.42 (14); and also similar to values for general implant surgeries, reported as 0.39 (SD 0.07) (25). Although this type of intervention is an invasive treatment, when compared to other dental procedures, it appears to cause less intraoperative pain. Other studies have reported mean VAS values during dental procedures as 3.1 (SD 2.8) for dental extractions (26), 2.9 (SD 3) for endodontic treatment (27), and 1.73 (SD 1.3) for supragingival scaling of the anterior six mandibular teeth without anesthesia (28).

As has been reported in other studies on dental implants, previous anxiety level (23), gender (25), age (23,25), operator skills (26), and tooth to be replaced (23) were not associated with patient reporting greater global pain through the VAS in dental implant surgeries in this study, considering the whole population.

The possibility of feeling pain during drilling or implant placement steps under nerve block is practically zero, and the possibility of it happening under mandibular infiltration is approximately one in ten implants placed. Pain experienced was variously reported as acute, short-termed, ceasing when stimulus ceased, low intensity, weak and bearable; and easily resolved by applying more anesthesia (MPQ-SV = 11 SD 4). Comparison of pain experiences under mandibular infiltration during these surgical steps (n=17) to painless surgeries (n=130) shows no statistical association with distance from the MC. Mean distance in the pain group was 3.8 (SD 2.2), and mean distance in the painless group was 3.00 (SD 1.8) (*p*=0.10). Also, when working closer than 2mm to the MC (traditional suggested safety zone), there were fewer painful than painless surgeries, with only 3 (9%) out of 33 implant placements feeling pain.

The reasons for pain during drilling under mandibular infiltration technique may be the distribution of the IAN in edentulous mandibles, the presence of both adjacent teeth, and the duration of the surgery.

In a cadaveric study on edentulous mandibles, IAN arrangement was found as a plexus inside the mandible in 96.3% of the sample, and as a single trunk in only 3.7% (13). When teeth are extracted, nerves degenerate but do not completely disappear. These nerve endings and neuromas were found in edentulous mandibles in animals, which were mechanically and thermally stimulated (29). In the present trial, these IAN branches, which may be far from the MC, may have been stimulated, producing pain.

Teeth adjacent to dental implant surgeries may contribute to presence of pain during these steps under mandibular infiltration. In this study, pain was significantly higher when both adjacent teeth were present than in surgeries performed next to only one or no adjacent tooth. This may be due to the mechanical stimulation of nerve endings of the periodontal ligament, which has not been reported previously.

Longer surgeries have been found to be significantly associated with intra-surgical pain in dental extractions (26). In the present study, prolonged surgery was also a factor associated with more chances of pain during drilling or implant placement under mandibular infiltration, enabling a statistical logistic model to be developed. The surgeon should pay special attention when applying this technique if the surgery lasts for a long period of time.

In this clinical trial, posterior mandibular bone was stimulated without working near the MC under mandibular infiltration anesthesia, as has been suggested by other authors (8,14,15,30). If painful cases are independent of distance to the MC, pain could occur at 1, 3, 5, or even 7mm away from it. If this pain is wrongly associated with being close to the MC, the professional might place a shorter implant, losing bone anchorage, or worse, unnecessarily aborting the surgery. When a painful situation occurs it is important that the surgeon should reinforce anesthesia, probe the walls of the implant site and take a periapical radiograph to evaluate distance to the MC. If sufficient distance from the nerve is confirmed, it is recommended to proceed with the surgery as planned originally. If the clinician is unsure of whether the MC has been perforated, it is recommended to abort the surgery.

## Conclusions

As global pain VAS values for mandibular infiltration technique were statistically higher than those for nerve block, and pain during drilling or implant placement under infiltration was significantly associated to longer surgical times, it is recommended to apply the nerve block technique when surgery is expected to be prolonged.

Pain should not be considered as a warning symptom to prevent nerve injuries in posterior mandible implant surgeries; since the proximity to the mandibular canal does not increase pain levels under mandibular infiltration technique.
